# Long-Term Excess Nitrogen Fertilizer Reduces Sorghum Yield by Affecting Soil Bacterial Community

**DOI:** 10.3390/plants15010025

**Published:** 2025-12-21

**Authors:** Qiuyue Wang, Juan Huang, Yaqin Zhang, Zebi Li, Ling Wei, Xuewei Yin, Xiaochun Zhang, Yu Zhou

**Affiliations:** Chongqing Academy of Agricultural Sciences, Chongqing 401329, China; wqy_825@163.com (Q.W.);

**Keywords:** long-term nitrogen fertilizer, sorghum, yield, soil properties, bacterial community

## Abstract

The application of nitrogen (N) fertilizer is one of the most important measures to affect crop yield and soil bacterial communities. In this study, the four rates of N (namely N0F 0 kg N ha^−1^, N1F 90 kg N ha^−1^, N2F 180 kg N ha^−1^, and N3F 270 kg N ha^−1^) along with a control (no fertilization, CK) were evaluated for their influence on sorghum yield, soil chemical properties, bacterial community, and diversity. The results showed that the yield-increasing effect was reduced by the higher dose of N input. Compared with N0F, sorghum yield increased by 58.8% in N1F and 68.2% in N2F but decreased by 8.1% in N3F relative to N2F. The soil pH decreased significantly with increasing N application. Compared with CK or N0F, N3F treatment increased the available P content by up to 18.6% or 32.2% but decreased the alkaline hydrolysis N, available K, organic matter, and total N contents by 8.4% or 23.8%, 5.5% or 10.6%, 8.4% or 28.8%, and 11.1% or 39.6%, respectively. In addition, different fertilization treatments altered the soil bacterial communities. Excess N fertilizer led to a decrease in bacterial abundance, and compared with N0F, the absolute abundance of bacteria increased by 18.7% in N1F, while it decreased by 31.8% in N3F. The predominant phyla, including Acidobacteria, Proteobacteria, and Chloroflexi, in the microbiome shift under different N application levels. The redundancy analysis (RDA) and Pearson’s correlation analyses indicated that the soil properties, especially soil pH, available P, total P, total N, and organic matter, were the key environmental factors that defined the bacterial community in the ecosystem. Within the scope of the present experiment, N application at 90 kg N ha^−1^ (N1F) optimized soil bacterial community abundance in sorghum-cultivated soil, while N2F (180 kg N ha^−1^) achieved the highest sorghum yield, suggesting a trade-off between optimizing the soil microbiome and maximizing crop yield under long-term fertilization.

## 1. Introduction

Nitrogen (N) is an essential nutrient element for the growth and development of plants and has a significant impact on crop growth and soil fertility [1,2,3]. In agricultural production, the application of chemical N fertilizer is one of the most rapid and most effective ways to increase grain yield [4,5]. The rational application of N fertilization has a clear and important relationship with sustainable crop production [6,7].

Due to China’s limited cultivatable land resources, the addition of excessive N fertilizers to boost aboveground biomass is a common agricultural practice in production [8]. Large quantities of N fertilizers (frequently >100 kg N ha^−1^) are applied directly to farmland ecosystems through anthropogenic activities each year [9,10]. Long-term application of chemical N fertilizer affects the soil quality, plant diversity, and productivity in terrestrial ecosystems by altering the soil physiochemical characteristics [11,12] and abundance and community structure of functional microorganisms [13,14,15]. Soil microbial communities are critical mediators of nutrient cycling and soil health [16,17], making their response to N fertilization key to understanding sustainable crop production [17,18]. Indeed, over the past decade, several studies have highlighted the influence of N addition on the soil microbial community. Xing et al. (2024) [19] proposed that short-term N enrichment (experimental duration ≤ 5 years) decreased the soil microbial biomass of natural grasslands, forests, and croplands. Wang et al. (2023) [20] showed that N addition significantly reduced soil bacterial diversity and shifted microbial community structure. Moreover, excess N fertilizer also reduced N_2_ fixation and signal transduction, the latter of which may contribute to the decreased interactions between bacteria and fungi [21].

High-throughput sequencing has greatly expanded the understanding of bacterial communities in different ecosystems, but conventional 16S rRNA gene sequencing can only detect the relative abundance of different microbial taxa found in a given sample and cannot obtain information on absolute microbial abundance, and it does not provide accurate information on the differences in abundance of target microorganisms between different samples [22]. Recently, the absolute quantification of microbial 16S rRNA sequencing (16S-seq) methods was performed by adding a synthetic spike-in standard sequence to sample DNA, constructing and sequencing 16S rRNA amplicon libraries, and simultaneously obtaining both the absolute and relative abundance of soil bacterial communities. The spike-in represents artificial 16S rRNA fragments that are designed in silico as variable regions that lack identification with nucleotide sequences in public databases, allowing reliable tracking of spike-in sequences in 16S-seq data from any microbiome sample [22]. Therefore, a comprehensive approach of adding a DNA internal standard to soil or other environmental samples prior to DNA extraction has been demonstrated as an effective method for comparing absolute bacterial cell abundances and community composition [22,23].

Sorghum (*Sorghum bicolor* (L.) Moench) is the world’s fifth major cereal crop after wheat, rice, corn, and barley [24]. It is considered an important crop in many areas, especially in tropical and semi-arid areas of the world due to its drought and barren tolerance ability [25]. N is the main nutrient for C4 plant productivity and plays a critical role in cell division during sorghum growth [26]. Previous research on N fertilization in sorghum-growing ecosystems has focused on yield and quality [27,28] and nitrogen-use efficiency [29], while little is related to molecular biology and microbiome responses under different levels of long-term N fertilizer additions, especially regarding changes in bacterial community structure and abundance.

In this study, we hypothesized that N application rates exceeding 180 kg ha^−1^ would decrease bacterial alpha diversity, shift community composition toward copiotrophic taxa, and reduce sorghum yield due to soil acidification and microbial dysfunction. We further hypothesized that a moderate N application rate would optimize the soil bacterial community, thereby supporting sustainable yield. The objectives of this study were (1) to evaluate the impact of long-term N fertilization on sorghum yield, soil properties, and the absolute abundance/diversity of the soil bacterial community, and (2) to explore the correlations between the bacterial community, soil chemical properties, and sorghum yield under different N levels.

## 2. Results

### 2.1. Sorghum Growth Characteristics and Yield Components

The sorghum yield in different treatments ranged from 3205.56 to 5954.17 kg ha^−1^. It is worth noting that N application significantly increased the sorghum yield (Table 1). Compared with N0F, the sorghum yield from the N-treated plots significantly (*p* < 0.05) increased by 54.6–68.2%, and the highest sorghum yield was recorded in N2F treatment. Sorghum yield under the N3F treatment was significantly lower by 8.1% (*p* < 0.05) compared to that under N2F, while no significant difference was observed between N3F and N1F. Moreover, the sorghum plant height, ear length, and ear grain weight were also significantly increased by N application, but N had no significant effect on 1000-grain weight. However, there were no significant differences in plant height, ear length, 1000 grain weight, and ear grain weight of sorghum between the three N application treatments.

### 2.2. Soil Properties in Field Experiments

The yield reduction under N3F was concomitant with severe soil degradation (Table 2). Soil pH decreased significantly with increasing N application, reaching strong acidity (pH 4.74) in the N3F treatment. The pH, AN, AK, OM, and TN of soil in the N3F treatment were significantly (*p* < 0.05) lower than those in other treatments, indicating a profound nutrient imbalance. Under different N levels, the highest OM was recorded in N0F (*p* < 0.05). Compared with CK or N0F, N3F treatment increased the AP content by up to 18.6% or 32.2% but decreased the AN, AK, OM, and TN contents by 8.4% or 23.8%, 5.5% or 10.6%, 8.4% or 28.8%, and 11.1% or 39.6%, respectively.

### 2.3. Bacterial Communities in Different Fertilized Soils

**Figure 1:**
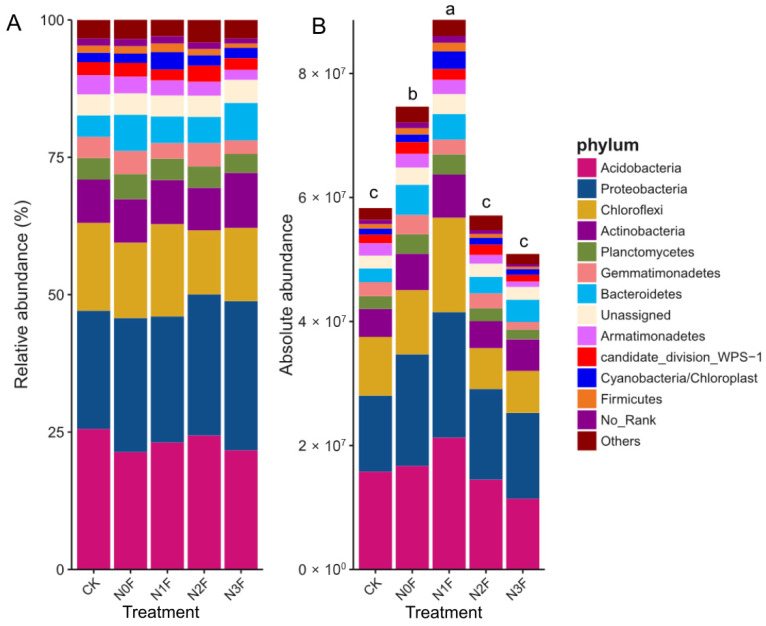
Relative abundances (**A**) and absolute abundances (**B**) (16S rRNA gene copies per g of soil) of the major bacterial phyla present in different N application rates of the soil samples. N0F, 0 kg N ha^−1^; N1F, 90 kg N ha^−1^; N2F, 180 kg N ha^−1^; N3F, 270 kg N ha^−1^. CK: the complete blank control with no fertilization. Different letters above the stacked bars indicate significant differences (*p* < 0.05) in total copy number between treatments.

Analysis of the soil bacterial community at the phylum level revealed that its structure and abundance were significantly influenced by N application. The community was consistently dominated by Acidobacteria (24.16%), Proteobacteria (23.96%), and Chloroflexi (14.69%), followed by Actinobacteria, Bacteroidetes, and Planctomycetes, among others (Figure 1A,B).

Crucially, the absolute abundance of the entire bacterial community exhibited a unimodal response to N fertilization, peaking significantly (*p* < 0.05) in the N1F treatment (Figure 1B). Compared to N0F, absolute bacterial abundance increased by 18.7% in N1F and by 52.0% compared to CK. This pattern was mirrored by the three dominant phyla, which also showed their highest abundances at N1F. In stark contrast, the N3F treatment harbored the lowest total bacterial abundance, representing a 31.8% decrease compared to N0F. This suppression of bacterial abundance under N3F directly paralleled the observed yield reduction, suggesting a potential functional link.

**Figure 2:**
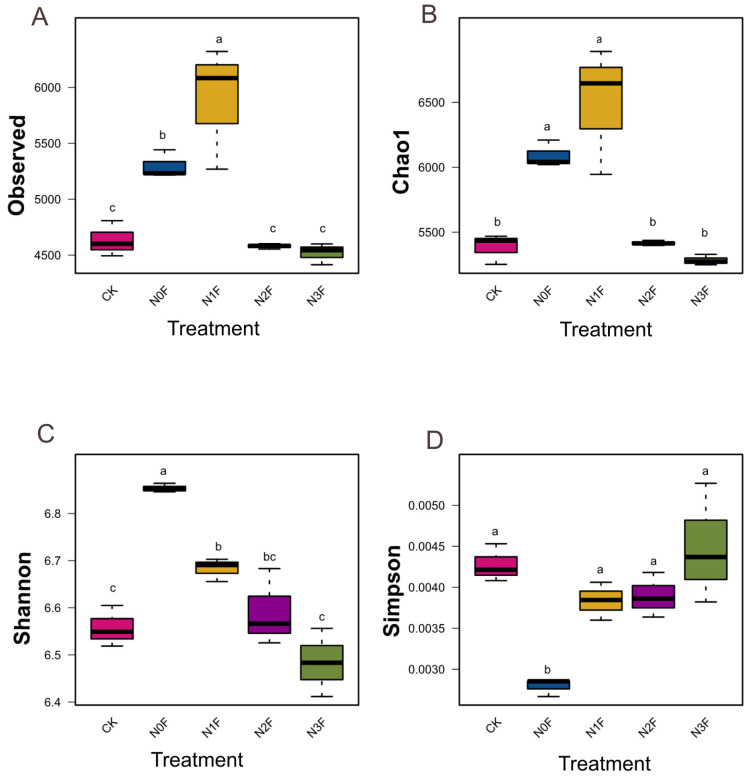
Effect of different fertilization on Alpha diversity ((**A**). Observed species, (**B**). Chao1, (**C**). Shannon, and (**D**). Simpson) of soil microbial community. N0F, 0 kg N ha^−1^; N1F, 90 kg N ha^−1^; N2F, 180 kg N ha^−1^; N3F, 270 kg N ha^−1^. CK: the complete blank control with no fertilization. Boxes with different letters are statistically significantly different; ANOVA, *p* < 0.05.

This trend was further supported by the alpha-diversity index. Both richness (observed species and Chao1) and diversity (Shannon index) generally decreased with increasing N application, indicating that high N fertilization simplifies the bacterial community (Figure 2A–C). The N1F treatment consistently supported the highest richness, while the indices for N2F and N3F were significantly lower than N0F. However, there was no significant difference in Simpson’s index among the various N application treatments (Figure 2D). The lack of significant change suggests that N fertilizer altered species richness and Shannon diversity more profoundly than community evenness in this system.

Furthermore, the Pearson correlation analysis results (Table 3) revealed a significant negative correlation between sorghum yield and the Shannon index (r = − 0.634, *p* = 0.010), indicating that higher yields were associated with lower bacterial diversity. Similarly, a significant positive correlation was observed between yield and the Simpson index (r = 0.585, *p* = 0.021), suggesting that higher yield corresponded to greater bacterial dominance (i.e., lower evenness). In contrast, no significant correlations were found between yield and richness index (observed species and Chao1). These findings imply that yield variation under long-term N fertilization is more closely linked to shifts in bacterial community structure (diversity and dominance) rather than to changes in species richness alone.

**Figure 3:**
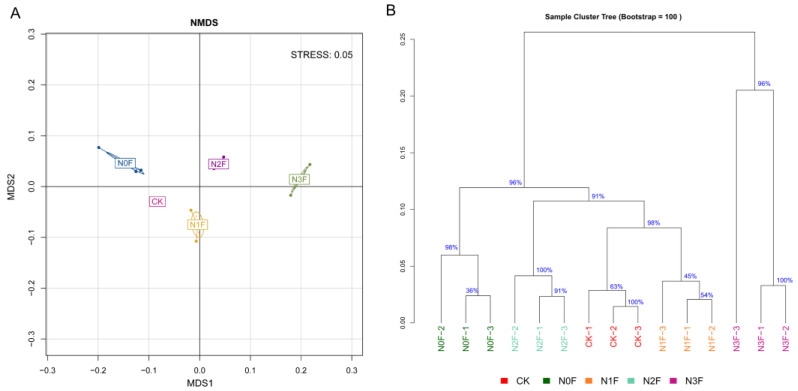
Non-metric multidimensional scaling (NMDS) (**A**) and hierarchical cluster tree (**B**) of bacterial communities (Bray–Curtis). N0F, 0 kg N ha^−1^; N1F, 90 kg N ha^−1^; N2F, 180 kg N ha^−1^; N3F, 270 kg N ha^−1^. CK: the complete blank control with no fertilization.

According to the results of non-metric multidimensional scaling (NMDS, Bray–Curtis, Figure 3A), the bacterial community of different treatments was undoubtedly separated. Compared with the CK, the soil bacterial communities were significantly altered by different fertilizer applications, and N input was the key factor affecting the soil bacterial community. The results of average linkage hierarchical clustering (calculated with Bray–Curtis, Figure 3B) showed that the bacteria of CK, N0F, N1F, and N2F were closer together, while the N3F was set apart, indicating that the bacterial community structure of N3F differed from the other treatments. It supported the results of NMDS.

**Figure 4:**
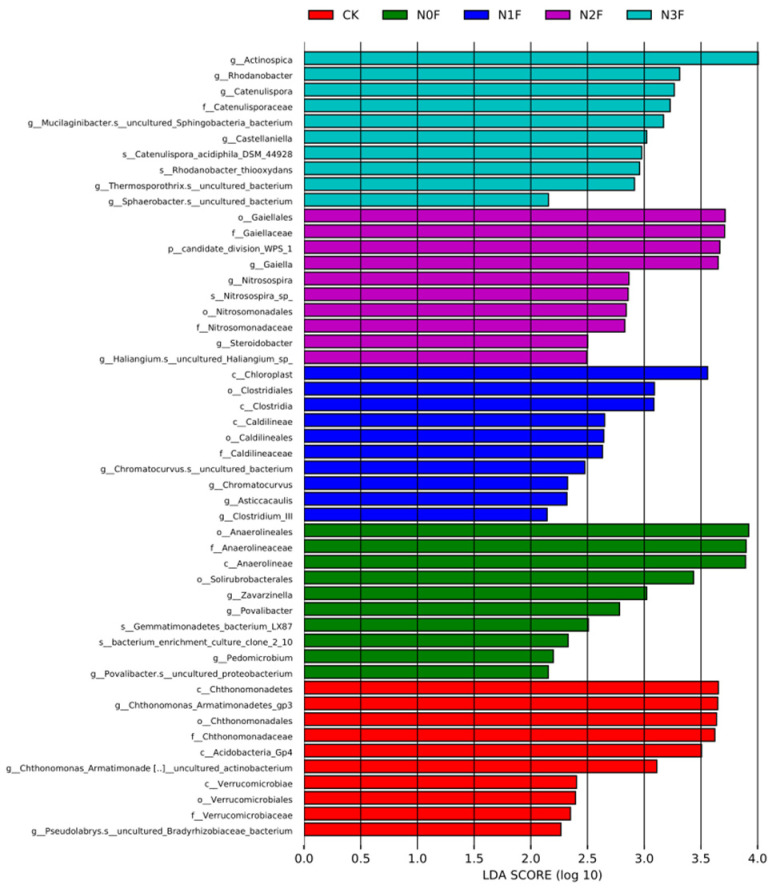
The linear discriminant analysis effect size (LEfSe) for significantly different abundant taxa of bacteria in different treatments. N0F, 0 kg N ha^−1^; N1F, 90 kg N ha^−1^; N2F, 180 kg N ha^−1^; N3F, 270 kg N ha^−1^. CK: the complete blank control with no fertilization.

LEfSe analyses were used to evaluate the bacteria, for which the abundance and copy number were significantly different in each treatment, and to analyze the biological relevance of the species. Linear discriminant analysis (LDA) scores higher than 2 were chosen to identify bacterial groups with statistically significant differences (Figure 4). The results of LEfSe found species that differed significantly between different treatments, with changes in Proteobacteria and Thaumarchaeota occurring mainly under high-N treatments (N3F), while changes in Chloroflexi and Firmicutes were primarily observed in N1F treatments. Among them, *Rhodanobacter* was more abundant under the N3F condition, whereas the order clostridia showed an increase under the N1F condition. The red color represents a significantly changed taxon in the CK, including Acidobacteria, Armatimonadetes, and Elusimicrobia, while Planctomycetes and Latescibacteria, represented in the green color, are species that differ significantly in abundance between N0F treatments.

**Figure 5:**
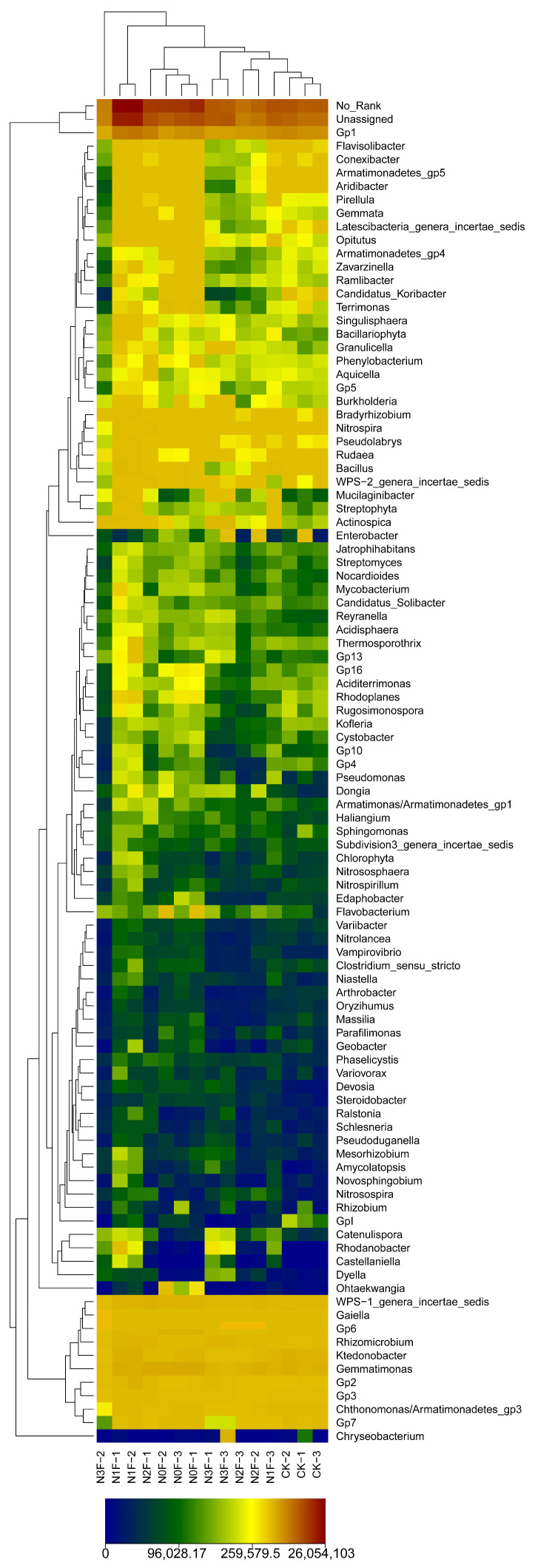
Heatmap of the distributions of the bacterial genera present in the soil samples of sorghum with different levels of N application. The absolute abundances of 100 bacterial genera in the soil samples are indicated by color intensity. The color gradient from blue to red indicates the species abundance from small to large. N0F, 0 kg N ha^−1^; N1F, 90 kg N ha^−1^; N2F, 180 kg N ha^−1^; N3F, 270 kg N ha^−1^. CK: the complete blank control with no fertilization.

The heatmap clustered samples based on distance, reflecting the similarity and specificity of species composition of all treatment samples at a given taxonomic level. As shown in Figure 5, the N1F treatment clustered into one category, while the other N application treatments were clustered into one category. The differences in N fertilization levels resulted in different absolute abundances of most of the selected genera (top 100 genera) in the treatments. The changes in color intensity exhibited that with the increase in N fertilizer application, the absolute abundance of species increased first and then decreased, which was shown as N1F > N0F > CK > N2F > N3F. Specifically, the soil of the N1F exhibited larger bacterial community abundance than that of other treatments, as shown with more orange and red columns by the color intensity.

### 2.4. Relationships Between the Community of Soil Microbiome and the Chemical Properties

**Figure 6:**
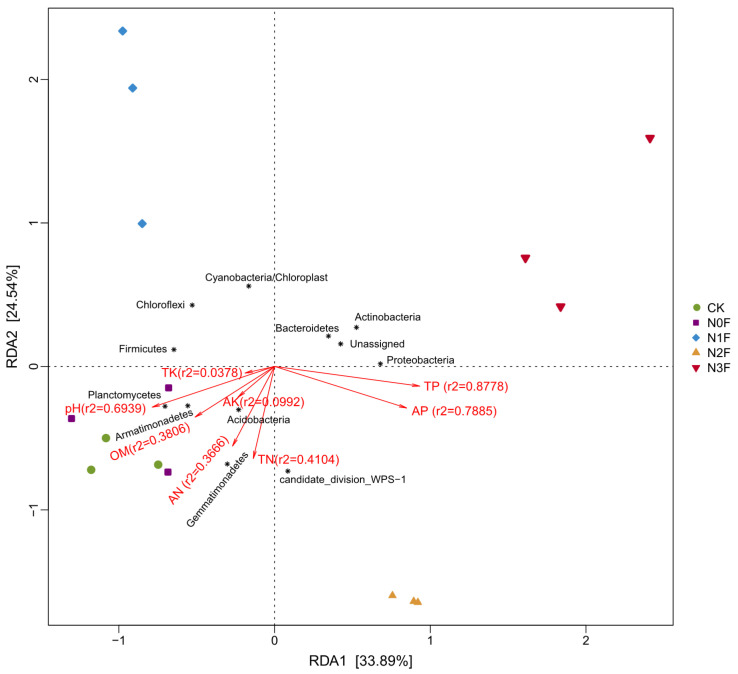
Redundancy analysis (RDA) of soil bacteria community with selected environmental variables for different N fertilizer treatments. N0F, 0 kg N ha^−1^; N1F, 90 kg N ha^−1^; N2F, 180 kg N ha^−1^; N3F, 270 kg N ha^−1^. CK: the complete blank control with no fertilization. AN, alkaline hydrolysis N; AP, available P; AK, available K; OM, organic matter; TN, total N; TP, total P; TK, total K.

The first component (RDA1) explained 33.89% of the variation in bacterial community structure and separated the N2F- and N3F-treated soil samples from the others (Figure 6). The second component (RDA2) separated the N1F and N3F treatments from the other treatments and explained 24.54% of the variation, indicating that the environmental factors selected for this study better explained the soil bacterial community in sorghum fields with different N applications. According to the results of the RDA, the contents of pH (r^2^ = 0.6939) and TN (r^2^ = 0.4104) were found to be positively correlated with most of the dominant phyla, whereas TP (r^2^ = 0.8778) and AP (r^2^ = 0.7885) were negatively correlated with the bacterial community. Of these, soil pH and TN had a greater effect on soil bacterial community structure in the CK treatment relative to the other treatments, whereas soil P, including TP and AP, had the greatest effect on bacterial community composition in the N3F.

The Pearson correlation coefficients (Table 4) showed that both soil AP and TP content were significantly negatively correlated to the abundance of Acidobacteria (*p* < 0.05) and Armatimonadetes (*p* < 0.01), respectively. The absolute abundances of Chloroflexi and Planctomycetes were significantly negatively correlated with soil AP content (*p* < 0.05), and the abundance of Gemmatimonadetes was significantly positively correlated with soil OM content (*p* < 0.05).

## 3. Discussion

N fertilizer is a cornerstone of modern agriculture, profoundly influencing crop productivity and soil microbial dynamics [19,20]. Our study demonstrates that the application of N fertilizer significantly enhanced sorghum yield, yet this effect diminished at higher application rates, with the highest yield observed at 180 kg N ha^−1^ (N2F). This aligns with the law of diminishing returns in crop response to N input, a phenomenon widely reported in cereal production systems [30,31]. The initial yield increase can be attributed to N’s critical role in chlorophyll synthesis, photosynthesis, and cell division, which are particularly crucial for a C4 plant like sorghum [32]. A critical finding was that when the N rate was increased to 270 kg N ha^−1^, grain yield significantly declined, becoming statistically indistinguishable from that under the 90 kg N ha^−1^ treatment. We posit that this nonlinear response indicates the existence of an optimal N threshold, beyond which multiple detrimental effects are triggered. The yield reduction under the highest N rate (N3F) suggests potential negative impacts such as nutrient imbalance [33], soil acidification [34,35], or microbial dysfunction [36,37], which may ultimately constrain plant growth. Collectively, these stressors underscore the instability and negative effects of supra-optimal N application—it not only fails to provide a yield advantage but effectively reverts productivity to a level comparable with N deficiency, while likely increasing performance variability. In contrast, the 180 kg N ha^−1^ treatment presumably represented the optimal N supply point under our experimental conditions, sufficiently meeting sorghum demand without inducing the severe physiological and edaphic stresses associated with N excess.

Concomitant with yield responses, long-term N fertilization markedly altered soil chemical properties. A consistent decrease in soil pH with increasing N application was observed, a common consequence of nitrification and the release of H^+^ ions from urea hydrolysis [38]. Notably, the highest N rate (N3F) led to significant reductions in AN, AK, OM, and TN compared to the no-N control (N0F) or moderate N treatments. This indicates that excessive N input may accelerate the decomposition of soil OM, promote leaching of base cations, and disrupt nutrient cycling [1,37]. The highest available P content in N3F likely resulted from reduced sorghum P uptake due to growth inhibition under strongly acidic soil conditions (pH 4.74), coupled with decreased microbial activity that would normally immobilize P in microbial biomass. This suggests that high N-induced acidification not only limits crop growth but also alters P dynamics in the soil–plant–microbe system [1].

The structure and abundance of the soil bacterial community were highly sensitive to N fertilization regimes [39]. The application of a moderate N rate (90 kg N ha^−1^, N1F) resulted in the highest bacterial abundance and richness, as evidenced by the Chao1 and observed species index. This suggests a fertilization effect that alleviates N limitation for microbial growth, thereby stimulating bacterial proliferation [40]. However, higher N inputs (N2F and N3F) suppressed bacterial diversity and absolute abundance. This decline is consistent with global trends of reduced microbial biomass under N enrichment [41] and can be explained by multiple factors: soil acidification directly inhibits many acid-sensitive bacterial groups [42], and increased osmotic stress from N salts can be detrimental [43].

Shifts in the predominant bacterial phyla further elucidate the impact of N fertilization. The dominance of Acidobacteria, Proteobacteria, and Chloroflexi across treatments is typical for agricultural soils [21,44]. Acidobacteria are known for degrading complex organic compounds such as cellulose and lignin, contributing to soil organic matter turnover and carbon sequestration [45,46]. Proteobacteria include many copiotrophic groups involved in nitrogen cycling (e.g., nitrification, denitrification) [47]. Chloroflexi are often associated with oligotrophic conditions and may play a role in carbon fixation [48]. The decline in Acidobacteria and Chloroflexi under high N (N3F) could reduce soil organic matter decomposition and nutrient mineralization, thereby limiting nutrient availability for sorghum. The LEfSe, heatmap, and NMDS analyses revealed that the bacterial community under the highest N input (N3F) was distinctly separated from other treatments. Specifically, high N favored certain Proteobacteria and Thaumarchaeota, which often include nitrifiers and opportunistic r-strategists thriving in resource-rich environments [49,50]. *Rhodanobacter* (a denitrifier) increased under N3F, suggesting enhanced denitrification potential under high N, and it dominated at low pH [51]. In contrast, the moderate N1F treatment was characterized by a significant increase in Chloroflexi and Firmicutes, phyla containing many heterotrophs involved in organic matter decomposition [52,53]. Clostridiales (anaerobic and capable of N fixation) were enriched in N1F, indicating balanced N conditions conducive to microbial N retention [54]. The decline in Acidobacteria under high N rates is likely a combined result of soil acidification beyond their optimal range and a shift in the soil environment towards a more copiotrophic state that disadvantages these oligotrophs [55]. These taxonomic shifts underscore how N application can restructure the microbial community by selecting for taxa with specific life-history strategies and nutrient acquisition mechanisms. Correlation analyses further elucidated the link between bacterial diversity and sorghum productivity. This pattern indicates that under optimal N fertilization (N2F), the soil bacterial community may become more functionally streamlined, with certain key taxa becoming dominant and potentially more efficient in supporting plant growth [55]. Such a shift could reflect a trade-off between microbial diversity and functional specialization, where a simplified but more efficient microbiome enhances nutrient cycling and plant performance [55,56].

The driving forces behind these microbial community changes were predominantly linked to key soil chemical properties [57]. RDA and correlation analyses identified soil pH, AP, TP, TN, and OM as the primary environmental factors shaping the bacterial community. The strong correlation between soil pH and bacterial composition is well-established, as pH influences enzyme activity, nutrient solubility, and the physiological limits of most bacteria [44,58]. Bacterial community composition showed nonlinear correlations with soil pH, an effect likely driven by the strong association of pH with other determinants like OM and AP. The negative associations between AP/TP and the abundance of several phyla, including Acidobacteria and Chloroflexi, suggest that P availability, likely modulated by N-induced acidification, is a critical factor in this ecosystem [1,14]. Furthermore, the positive correlation of OM with bacterial groups like Gemmatimonadetes highlights the importance of carbon availability in sustaining a diverse and abundant microbiome [59]. This intricate web of interactions confirms that the bacterial community response to N fertilization is not direct but mediated through a cascade of changes in the soil’s physicochemical environment. Overall, long-term N fertilization significantly reduced soil pH, which, in turn, decreased bacterial diversity and shifted community composition toward acid-tolerant but less diverse taxa. These microbial changes likely impaired nutrient cycling functions, contributing to the observed yield reduction under high N input (N3F).

This study has several limitations: (1) it was conducted at a single location with one sorghum variety, limiting generalization; (2) only bacterial communities were examined, while archaea, fungi, and other microbiota may also respond to N fertilization; and (3) the experiment lacked organic or integrated nutrient management comparisons, which are important for sustainable agriculture. Future studies should include multi-location trials, multi-kingdom microbial analyses, and organic–inorganic fertilizer comparisons.

## 4. Materials and Methods

### 4.1. Experimental Site and Climatic Conditions

**Figure A1:**
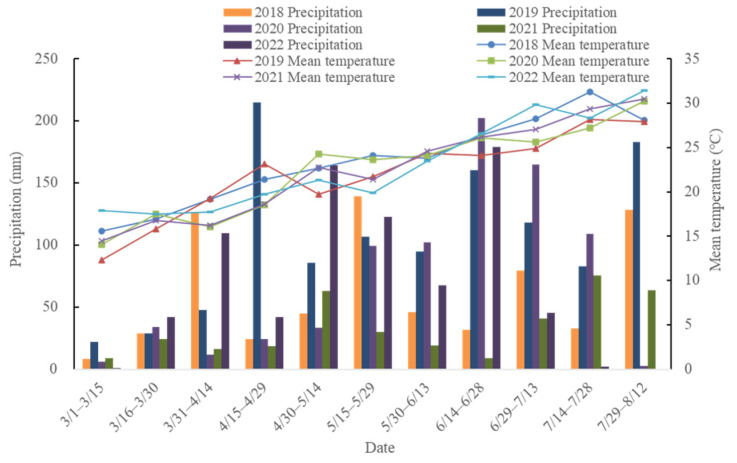
Precipitation and daily mean temperature during the sorghum-growing season (2018–2022).

The experimental site was located at the Western Crop Experiment Station of Chongqing Academy of Agricultural Sciences in Wujian Town, Yongchuan District, Chongqing, China (29°10′28″ N, 105°49′13″ E). The soil at the experimental site is classified as an Alisol (World Reference Base for Soil Resources). The area enjoys a typical subtropical monsoon humid climate, with a mean annual temperature of 17.7 °C, a mean annual precipitation of 1015.0 mm, a mean annual sunshine of 1218.7 h, and a frost-free period of 317 days. Meteorological data (including daily mean temperature and precipitation) for the duration of the sorghum-growing season (March to August) in the experimental year (2018–2022) were collected from a local weather station located approximately 5 km from the test site and are presented in Appendix A Figure A1. The mean annual sunshine duration from 2018 to 2022 was 1255.4 h. The soil of each treatment plot was clay-textured with uniform fertility. Prior to the initiation of the fertilization treatments in 2018, initial soil samples were collected and analyzed. The soil alkaline hydrolysis N (AN), available P (AP), available K (AK), organic matter (OM) content, and pH value were 61.42 mg kg^−1^, 8.16 mg kg^−1^, 93.12 mg kg^−1^, 10.68 g kg^−1^, and 5.75, respectively.

### 4.2. Field Experiment Design

The field experiment was carried out in a continuous sorghum cropping area from 2018 to 2022. The field was rainfed, and no additional irrigation was applied during the growing season. Standard agronomic practices for weed and pest control were uniformly applied across all plots to minimize their impact on the experiment. The sorghum variety used was Jinyunuo 3, a locally adapted and high-yielding glutinous sorghum cultivar provided by the Chongqing Academy of Agricultural Sciences, China, selected for its suitability to the regional growing conditions and market demand. This experiment was carried out in a randomized block design with four nitrogen (N) fertilizer rates, including (1) no N, 0 kg N ha^−1^ (N0F), (2) N deficiency, 90 kg N ha^−1^ (N1F), (3) recommended N rate for sorghum, 180 kg N ha^−1^ (N2F), (4) supra-optimal levels, 270 kg N ha^−1^ (N3F), and no fertilization as a complete blank control (CK), which received no N, phosphorus (P), or potassium (K) fertilizers. Each treatment was replicated three times, and every plot was allocated an area of 4 m × 6 m. The application rates of conventional fertilizers were 90 kg P_2_O_5_ ha^−1^ (calcium superphosphate, 12% P_2_O_5_) and 75 kg K_2_O ha^−1^ (potassium chloride, 60% K_2_O), except for CK. N fertilizer (urea, 46% N) was applied according to the ratio of basal fertilizer: jointing fertilizer at 6:4. P and K fertilizer was added by broadcasting as a single dose and mixed into the 0–20 cm plow layer of soil before sorghum sowing. The target planting density of 105,000 plants ha^−1^ was established by sowing seeds at a fixed spacing within rows (50 cm between rows and 38 cm between hills within the row, with 2 plants per hill) and was maintained by thinning seedlings at the 3–5 leaf stage.

### 4.3. Sample Preparation and Analyses

At maturity, the grain yield was estimated to be from a harvest area of 6 m^2^ within the central part of each plot to avoid border effects. The grains from all plants within this area were threshed, air-dried, and weighed to calculate the plot yield. Additionally, 20 representative plants were randomly selected from each plot to determine the plant height, panicle length, and yield components, including the average ear grain weight and 1000-grain weight. The final grain yield per hectare was calculated based on the plot yield and harvest area. Soil samples were collected at the harvesting stage of sorghum in August 2022, which represented the 5th year of N fertilizer application. A square area of 20 × 20 cm was measured on the sides of each plant root circumference of 10 cm and positioned as a sampling area. The top 20 cm of soil was sampled, with 5 cores of soil (4 cm diameter) taken at chosen points through each experimental plot and mixed into one sample as a replicate, with three replicates for each treatment. All samples were composited and sieved through a 2 mm screen to thoroughly homogenize them, and the roots, stones, and other debris were removed. A portion of each soil sample was collected in a 50 mL centrifuge tube and transferred to the laboratory. The tubes were stored at −80 °C for the extraction of DNA from the soil. The remaining soil samples were air-dried and sieved to determine the soil properties.

### 4.4. Soil Property Determination

Soil pH was measured using a pH meter (Seven Easy Mettler Toledo, Shanghai, China) after shaking the soil water slurry (1:2.5 *w*/*v*) suspension for 30 min. The soil total N (TN) content was analyzed using the Kjeldahl method [60]. Soil total P (TP) and total K (TK) were determined by NaOH molybdenum-blue colorimetry and flame photometry (FP-6410; Xinyi Instruments, Shanghai, China), respectively. AN in soil was extracted using the alkaline hydrolysis diffusion method. AP in soil was extracted by sodium bicarbonate and measured using the molybdenum-blue method [61] (UV-2450; Shimadzu, Kyoto, Japan). AK in soil was extracted by ammonium acetate and determined by flame photometry (Hitachi Z2000; Hitachi High-Tech Corporation, Tokyo, Japan). The OM content was assayed via the potassium dichromate volumetric method [62].

### 4.5. DNA Extraction and Absolute Quantification of 16S rRNA

Soil samples (0.8 g wet weight) collected from the 5 treatments (CK, N0F, N1F, N2F, and N3F) were taken for total genomic DNA extraction by using the MoBioPowerSoil DNA Isolation Kit according to the manufacturer’s instructions (MoBio, Carlsbad, CA, USA). The integrity of soil DNA was tested by 0.7% agarose gel electrophoresis. There were multiple different spike-in sequences with conserved regions identical to those of selected natural 16S rRNA genes, and the artificial variable regions were added to the sample DNA pools. A known concentration (1 × 10^6^ copies per reaction) of synthetic spike-in DNA (designed with variable regions absent in natural databases) was added to each soil DNA sample prior to PCR amplification. Spike-in recovery efficiency ranged from 85 to 95%, and only samples with recovery >80% were included in the absolute abundance calculation. Spike-in sequences were bioinformatically identified and removed after absolute quantification. The DNA samples were amplified with particular primers, 515F (5′-GTGCCAGCMGCCGCGG-3′) and 907R (5′-CCGTCAATTCMTTTRAGTTT-3′), which targeted the hypervariable V4–V5 region of the bacterial 16S rRNA gene. The PCR products were purified with a PCR Clean-UpTM kit (MO BIO Labs, Solana Beach, CA, USA), and then, they were sequenced with the Illumina Miseq platform (Genesky Biotechnologies Inc., Shanghai, China).

### 4.6. Illumina Read Data Processing and Analysis and Absolute Quantification of 16S rRNA

TrimGalore (version 0.4.5, http://www.bioinformatics.babraham.ac.uk/projects/trim_galore/ (accessed on 22 May 2023)), FLASH2 (version 2.2.00) [63], and Mothur (version 1.25.1, https://www.mothur.org/ (accessed on 23 May 2023)) were used to process the high-throughput sequencing data. Only the sequence length longer than N200 bp with an average quality score above Q20 was included in the subsequent analysis. The filtered sequences were clustered into 16S rRNA operational taxonomic units (OTUs) based on a 97% sequence similarity with UCLUST (version 4.2.40). The resulting OTU representation sequence was annotated. Spike-in sequences were removed after absolute abundance calculation but before relative abundance and diversity analyses. A standard curve of read counts versus spike-in the DNA copy number was established, and the absolute copy number of each OTU in each sample was calculated. The aligned sequences were classified by the databases of the Ribosomal Database Project (RDP), which was performed by Mothur (version 1.25.1). The soil bacterial abundance and diversity were analyzed considering OTU tables.

### 4.7. Statistical Analysis

Sorghum yield, soil properties, and microbial abundance data were analyzed by using one-way analysis of variance (ANOVA) through IBM SPSS 20.0 (IBM Corporation, Armonk, NY, USA). Pearson correlation analysis was employed to examine the relationships between soil bacterial alpha diversity and sorghum yield, as well as between the soil bacterial community and environmental factors. Redundancy analysis (RDA) was implemented in the R software (version 3.2.2) to elucidate the relationships between the soil bacterial community and chemical properties under the application of different levels of N fertilization. Non-metric multidimensional scaling (NMDS) analysis was sorted based on any type of distance/non-similarity matrix for the total bacterial community analyses [64]. The average linkage hierarchical clustering and heatmap were performed by R (version 3.2.2). Microbial communities were characterized in each of the N treatments using a linear discriminant analysis (LDA) effect size (LEfSe) approach, which emphasizes statistical significance and biological relevance.

## 5. Conclusions

The application of N fertilizer significantly increased sorghum yields, and different N applications altered the structure and diversity of the soil bacterial community. Moderate amounts of N fertilizer increased the abundance of soil bacteria and had a significant effect on dominant microbial species, thus improving the soil bacterial community structure, while in the meantime, excessive N fertilizer decreased soil bacterial abundance. The shift in soil bacterial community composition was mainly driven by soil chemical properties such as pH, available phosphorus (AP), total phosphorus (TP), organic matter (OM), and total nitrogen (TN). Notably, sorghum yield was significantly correlated with bacterial diversity index (Shannon and Simpson), highlighting the role of microbial community structure in mediating crop productivity under N fertilization. Among the treatments, 90 kg ha^−1^ N per year (N1F) was optimal for enhancing soil bacterial abundance, whereas 180 kg ha^−1^ N (N2F) was optimal for maximizing yield. This study provides a theoretical basis for reducing N fertilization and improving soil quality for a sustainable sorghum industry.

## Figures and Tables

**Table 1:** Effects of different N fertilizer treatments on the growth and yield composition of sorghum.

Treatment	Plant Height (cm)	Ear Length (cm)	1000-Grain Weight (g)	Ear Grain Weight (g)	Yield (kg ha^−1^)
CK	150.37 ± 10.92 ^b^	30.64 ± 2.41 ^b^	26.13 ± 1.12 ^a^	35.50 ± 2.92 ^b^	3205.56 ± 232.38 ^c^
N0F	154.53 ± 9.67 ^b^	31.77 ± 1.19 ^b^	26.62 ± 0.97 ^a^	38.93 ± 3.13 ^b^	3540.28 ± 231.14 ^c^
N1F	179.27 ± 1.22 ^a^	34.74 ± 0.59 ^a^	27.29 ± 2.07 ^a^	61.46 ± 3.52 ^a^	5622.22 ± 146.86 ^ab^
N2F	182.80 ± 2.04 ^a^	35.59 ± 0.62 ^a^	28.78 ± 2.63 ^a^	64.74 ± 3.29 ^a^	5954.17 ± 82.07 ^a^
N3F	184.00 ± 1.97 ^a^	35.61 ± 1.08 ^a^	28.36 ± 0.54 ^a^	64.71 ± 1.62 ^a^	5471.94 ± 197.50 ^b^

N0F, 0 kg N ha^−1^; N1F, 90 kg N ha^−1^; N2F, 180 kg N ha^−1^; N3F, 270 kg N ha^−1^. CK: the complete blank control with no fertilization. Means ± standard deviation (*n* = 3). Different letters indicate significant differences between fertilization treatments (ANOVA, *p* < 0.05).

**Table 2:** Effects of different N application levels on soil chemical properties.

Treatment	pH	AN (mg kg^−1^)	AP (mg kg^−1^)	AK (mg kg^−1^)	OM (g kg^−1^)	TN (g kg^−1^)	TP (g kg^−1^)	TK (g kg^−1^)
CK	5.44 ± 0.03 ^a^	44.82 ± 1.98 ^c^	10.46 ± 0.33 ^c^	96.23 ± 1.49 ^d^	10.29 ± 0.04 ^d^	0.36 ± 0.01 ^d^	0.33 ± 0.01 ^b^	20.60 ± 0.36 ^b^
N0F	5.37 ± 0.02 ^b^	53.86 ± 1.42 ^a^	9.39 ± 0.16 ^d^	101.70 ± 1.49 ^c^	13.24 ± 0.23 ^a^	0.53 ± 0.02 ^b^	0.32 ± 0.03 ^b^	19.94 ± 0.48 ^b^
N1F	4.93 ± 0.02 ^c^	48.58 ± 0.56 ^b^	8.64 ± 0.16 ^e^	109.66 ± 0.99 ^b^	10.77 ± 0.20 ^c^	0.40 ± 0.01 ^c^	0.30 ± 0.01 ^b^	22.13 ± 0.46 ^a^
N2F	4.82 ± 0.02 ^d^	56.30 ± 1.73 ^a^	11.28 ± 0.23 ^b^	113.64 ± 1.49 ^a^	11.23 ± 0.20 ^b^	0.57 ± 0.01 ^a^	0.39 ± 0.02 ^a^	22.04 ± 0.76 ^a^
N3F	4.74 ± 0.02 ^e^	41.05 ± 0.86 ^d^	12.41 ± 0.35 ^a^	90.92 ± 1.25 ^e^	9.43 ± 0.34 ^e^	0.32 ± 0.02 ^e^	0.42 ± 0.03 ^a^	19.60 ± 0.48 ^b^

N0F, 0 kg N ha^−1^; N1F, 90 kg N ha^−1^; N2F, 180 kg N ha^−1^; N3F, 270 kg N ha^−1^. CK: the complete blank control with no fertilization. AN, alkaline hydrolysis N; AP, available P; AK, available K; OM, organic matter; TN, total N; TP, total P; TK, total K. Means ± standard deviation (*n* = 3). Different letters indicate significant differences between fertilization treatments (ANOVA, *p* < 0.05).

**Table 3:** Pearson correlation coefficients between sorghum yield and soil bacterial alpha-diversity index under different nitrogen fertilization treatments.

Alpha-Diversity Index	Correlation Coefficient (r)	*p*-Value	Significance
Observed species	−0.433	0.103	NS
Chao1	−0.442	0.094	NS
Shannon	−0.634	0.010	**
Simpson	0.585	0.021	*

NS: not significant; *: *p* < 0.05; **: *p* < 0.01.

**Table 4:** Correlation between soil chemical properties and abundance of dominant soil bacterial phylum in sorghum soil.

Abundant Taxa	pH	AN	AP	AK	OM	TN	TP	TK
Acidobacteria	0.280	0.306	−0.956 *	0.557	0.360	0.136	−0.899 *	0.599
Proteobacteria	−0.079	0.375	−0.781	0.508	0.498	0.272	−0.572	0.366
Chloroflexi	0.240	0.024	−0.905 *	0.301	0.207	−0.137	−0.861	0.395
Actinobacteria	−0.030	0.002	−0.746	0.200	0.254	−0.109	−0.600	0.178
Bacteroidetes	0.011	0.185	−0.483	0.030	0.571	0.180	−0.294	−0.242
Planctomycetes	0.351	0.489	−0.958 *	0.503	0.701	0.372	−0.840	0.340
Gemmatimonadetes	0.618	0.790	−0.749	0.547	0.923 *	0.732	−0.703	0.264
Armatimonadetes	0.676	0.365	−0.965 **	0.378	0.590	0.231	−0.988 **	0.329
candidate_division_WPS-1	0.319	0.859	−0.782	0.745	0.877	0.782	−0.643	0.465

AN, alkaline hydrolysis N; AP, available P; AK, available K; OM, organic matter; TN, total N; TP, total P; TK, total K. “*” and “**” were significantly correlated at the *p* = 0.05 and 0.01 levels, respectively.

## Data Availability

The datasets generated for this study are available on request to the corresponding author.

## Abbreviations

The following abbreviations are used in this manuscript:

AN	Alkaline hydrolysis nitrogen
AP	Available phosphorus
AK	Available potassium
ANOVA	One-way analysis of variance
K	Potassium
LDA	Linear discriminant analysis
LEfSe	Linear discriminant analysis effect size
N	Nitrogen
NMDS	Non-metric multidimensional scaling
OM	Organic matter
OTU	Operational taxonomic unit
P	Phosphorus
RDP	Ribosomal Database Project
TK	Total potassium
TN	Total nitrogen
TP	Total phosphorus
